# Professionals’ attitudes towards people with intellectual disabilities who self-harm: A literature review

**DOI:** 10.1177/17446295211025959

**Published:** 2021-08-02

**Authors:** Beverley Samways

**Affiliations:** 1980 University of Bristol, UK

**Keywords:** attitudes, NICE, self-harm, self-injurious behaviour

## Abstract

**Background:**

National Institute for Health and Care Excellence (NICE, 2013) Guidance on Self-Harm states that professionals supporting people who self-harm should demonstrate compassion, respect and dignity. This literature review examines the evidence for professionals’ attitudes towards people with intellectual disabilities who self-harm.

**Method:**

Four databases (PsychInfo, IBSS, CINAHL and Medline) were systematically searched to find relevant research since 2000.

**Results:**

Four studies met the criteria. Attitudes of professionals supporting people with intellectual disabilities are contrasted with those of professionals in settings focused on supporting people without intellectual disabilities. Professionals supporting people with intellectual disabilities tended to display attitudes and attributions reflective of biobehavioural and psychosocial theories of self-harm, with a greater emphasis on relationships.

**Conclusion:**

Much more research is needed which examines the attitudes of professionals supporting people with intellectual disabilities who self-harm.

## Introduction

### Self-harm and self-injurious behaviour

Self-harm is defined by the National Institute for Health and Care Excellence (NICE) as, ‘any act of self poisoning or self injury carried out by a person, irrespective of their motivation’ (NICE Guidance on Self-harm, 2013: 6). Definitions of self-harm are sometimes separated into ‘suicidal self-harm’ and ‘non-suicidal self-harm’ (NSSH). For the purposes of this study, the term ‘self-harm’ is taken to mean non-suicidal self-harm.

Self-harm represents a significant challenge for public health in the UK (Evans et al., 2019; HM Government, 2019; Public Health England, 2017). In 2014, 32% of female and 11% of male 15-year olds reported that they had self-harmed (Public Health England, 2017). McManus et al. (2019) list the most common reason for self-harm as being ‘to relieve unpleasant feelings of anger, tension, anxiety, or depression’ (2019: 573); it is ‘strongly associated with emotional distress and mental health issues and…accompanied by a complex set of negative feelings such as self-loathing, disgust and shame’ (Public Health England, 2017: 6).

Both in and outside the UK, research has tended to separate self-harm presented by people with and without intellectual disabilities (Bradley et al., 2018; Richards and Symons, 2018). ‘Self-harm’ is the preferred term for people without intellectual disabilities, whilst ‘self-injurious behaviour’ has traditionally been the common term for those with intellectual disabilities (Heslop and Lovell, 2013; NICE Guidance on Challenging Behaviour, 2015). Rojahn et al. (2008) define self-injurious behaviour as a pathological behaviour (i.e. clinically significant), with repeated and largely uniform patterns, which ‘cause or have the potential to cause direct or indirect (cumulative) physical damage to the person’s own body’ (p. 2). The prevalence of self-injurious behaviour amongst people with intellectual disabilities varies from 4% to 24% and is generally very persistent (Oliver and Richards, 2015).

NICE (2015) incorporates guidance about self-injurious behaviour into its broader guidance on challenging behaviour and intellectual disabilities. This guidance differentiates self-harm from self-injurious behaviour, stating that self-harm is ‘when a person intentionally harms themselves, which can include cutting and self-poisoning’, whereas self-injury is ‘repeated, self-inflicted behaviour, such as people hitting their head or biting themselves’ (p. 29). Self-injurious behaviour typically presents in people with intellectual disabilities, and may indicate pain, distress or another purpose, such as communication (NICE Guidance on Challenging Behaviour, 2015).

There are distinct theories of causation for self-injurious behaviour: behavioural and biological theories have tended to dominate the literature (Chezan et al., 2017; Oliver and Richards, 2015). Operant learning is the prevailing behavioural theory (Symons et al., 2012). It states that the reason for self-injurious behaviour may initially be innocuous, such as a compulsion to communicate something or a display of anger or pain, but then the behaviour engenders a response that reinforces the behaviour (e.g. the person’s need is met unusually quickly or levels of attention or concern are heightened). Thus, self-injurious behaviour is ‘positively or negatively reinforced by sensory, tangible or social stimuli’ (Oliver and Richards, 2015: 1045). Functional analysis enables the identification of what is maintaining self-injurious behaviour and addresses it from a functional perspective (Hagopian et al., 2015); thus, behavioural management is the advocated approach for self-injurious behaviour (Symon et al., 2012). In addition, biological theories have found that self-injurious behaviour can be associated with a genetic condition or diagnosis, a response to pain or part of a movement disorder (Oliver and Richards, 2015). The underlying assumption is that self-injurious behaviour is not inherently meaningful for the individual (Favazza, 1992; Furniss and Biswas, 2012); this typically leads to pharmacological interventions (Rana et al., 2013; Read and Rendell, 2007). Thus, emotional distress and mental health problems, considered at the root of self-harm for people without intellectual disabilities, are much less likely to be considered, or are disregarded, for people with intellectual disabilities (Dick et al., 2011; Lovell, 2008).

The term ‘self-injurious behaviour’ aligns itself squarely with biobehavioural theory and practice (i.e. the combined application of behavioural and biological theories). However, there is a growing emphasis on the similarities found between self-harm amongst those with and without intellectual disabilities, particularly as pertains to the emotional function of self-injury (Dick et al., 2011; Ghaderi et al., 2017; Heslop and Lovell, 2013). This does not necessarily negate biobehavioural theory and practice but considers concurrently whether self-injurious behaviour may also be a communication of ‘distress’ (NICE Guidance on Challenging Behaviour, 2015).

Three out of four of the studies in this review from settings supporting people with intellectual disabilities adopt the term ‘self-harm’, maybe to reflect that their findings align more closely with the psychosocial factors (i.e. that self-harm is a response to emotional dysregulation or distress) than the biobehavioural foundation of ‘self-injurious behaviour’. This literature review similarly adopts the term ‘self-harm’ as an all-incorporating term; this follows the precedent of studies discussing similar themes (Lovell, 2008; Rees and Langdon, 2016).

### Attitudes

NICE Guidance on Self-Harm (2013) sets an expected standard for professionals’ attitudes towards those who self-harm, stating the importance of ensuring that ‘people who have self-harmed are cared for with compassion and the same respect and dignity as any service user’ (p. 10). This is paramount as ‘staff attitudes are often reported as contributing to poor experiences of care. Punitive or judgemental staff attitudes can be distressing for people who have self-harmed and may lead to further self-harm or avoidance of medical attention’ (NICE Guidance on Self-Harm, 2013: 10). In the same vein, staff attitudes and responses to people with intellectual disabilities who self-harm have been identified as reinforcing or exacerbating of the behaviour (Hastings et al., 2003; Heslop and Macauley, 2009).

Attitudes are classically defined by Allport (1935) as ‘a mental and neural state of readiness organized through experience, exerting a directive or dynamic influence upon the individual’s response to all objects and situations with which it is related’ (p. 810). Attitudes are the ‘evaluative judgments’ held which affect a person’s reactions (Crano and Prislin, 2006). Research examining attitudes frequently draws on Weiner’s (1986) attributional theory of motivation and emotion (Dick et al., 2011; Marzano et al., 2015), which proposes that people’s beliefs and assumptions about the cause of something are a key determinant of their emotional and behavioural responses (Reisenzein and Rudolph, 2018). Thus, research into professional attitudes has tended to measure professionals’ attributions about self-harm as well as their attitudes (Williams et al., 2012). Positive attitudes towards self-harm are largely considered within the literature to be compassion and empathy (Karman et al., 2015; Rayner et al., 2019), alongside understanding and a sense of confidence. Negative attitudes are associated with frustration, anger and hostility (Rayner et al., 2019), described as ‘punitive or judgemental’ (NICE Guidance on Self-Harm, 2013: 10) or brought under the umbrella term ‘antipathy’ (Patterson et al., 2007: 438).

Working with people who self-harm can be emotionally challenging, particularly for those who see themselves in a helping or healing role; this is found amongst professionals supporting people with intellectual disabilities (Fish and Duperouzel, 2008; Hastings et al., 2003) as well as in settings focused on supporting those without intellectual disabilities (Marzano et al., 2015; Patterson et al., 2007). The sometimes-conflicting emotions of feeling responsible to help whilst simultaneously feeling powerless to do so, can lead to professionals distancing themselves as a defence mechanism (Marzano et al., 2015) and placing the locus of the problem within the individual, rather than themselves or their skill (Huband and Tantam, 2000). Adequate training to improve staff understanding and regular supervision are routinely cited as pre-requisites for professionals supporting those who self-harm if they are going to maintain the compassion and respect required; there is a well-established efficacy for direct training improving knowledge, confidence and empathy (Karman et al., 2015; McHale and Felton, 2010; Rees et al., 2015).

Research has established that professionals’ attitudes to self-harm are significant to the maintenance^1^ of self-harm for both those with intellectual disabilities (Hastings et al., 2003) and those without intellectual disabilities (Timson et al., 2012). This literature review examines professionals’ attitudes and attributions towards people with intellectual disabilities who self-harm, contrasting them with reported attitudes and attributions of professionals from services not focused on people with intellectual disabilities. The aim is to identify and examine apparent differences in attitudes between professionals supporting people with and without intellectual disabilities who self-harm, contrasting the evidence from studies since 2000,^2^ with a focus on informing policy and practice in relation to professional support of people with intellectual disabilities who self-harm.

## Method

### Design

This review draws on the PRISMA guidelines (Moher et al., 2009). It entailed a systematic literature search and an iterative approach to thematic analysis (Braun and Clarke, 2006).

### Search method

PsychInfo, IBSS, CINAHL, Web of Knowledge and Medline databases were searched for peer-reviewed, UK studies since 2000. Three blocks of search terms were developed to capture the various synonyms, combining them with the Boolean operator ‘AND’ (Robertson et al., 2019). Terms for ‘self-harm’, ‘attitudes’ and ‘professionals’ were collated by examining the reference lists of articles already found and taking advantage of the alternatives suggested by the EBSCOhost database. Table 1 lists the search terms used. Articles prior to 2000 were excluded, as this review considers the definitions and requirements of the NICE Guidance on Self-Harm (2013), which were first established in 2000. Articles discussing both self-harm and suicide or conflating the two were excluded (e.g. McCann et al., 2007). Articles discussing only definitions or knowledge of professionals were excluded, as this review was interested specifically in attitudes (e.g. Simm et al., 2008). Also excluded were articles only concerned with attributional theories or models or the factors influencing attitudes, as this review was concerned with what the attitudes of professional were, not influencing factors for attitudes or attitudinal models (e.g. Hastings et al., 2003; Wheatley and Austin-Payne, 2009). Articles were also excluded if they focused on comparison, e.g. articles stating that attitudes were better or worse between professions, without saying what those attitudes actually were (e.g. Hastings et al., 2003; Mossman et al., 2002). Lastly, studies examining the attitudes of non-professionals, e.g. students or families (e.g. Fox, 2016), were excluded. This search was conducted in May 2019. See Table 2 for inclusion and exclusion criteria in full.

**Table 1.** Search terms.

Search term	Variation
Self-harm	‘self-injury’ OR ‘self-harm’ OR ‘self-mutilat*’ OR ‘self-injurious’
Attitudes	‘attitude*’ OR ‘perception*’ OR ‘opinion*’ OR ‘thoughts’ OR ‘feelings’ OR ‘belief*’ OR ‘reaction*’ OR ‘attribution*’
Professionals	‘professional*’ OR ‘staff’ OR ‘teacher*’ OR ‘support workers’ OR ‘carer*’

**Table 2.** Inclusion and exclusion criteria.

Inclusion criteria
– Peer-reviewed – Published from 2000-present – English language full text – Self-injury or self-harm (or equivalent terms) – Professionals’ (or equivalent terms) attitudes (or equivalent terms) towards self-harm – Quantitative, qualitative or mixed method studies
Exclusion criteria
– Not peer-reviewed or status unclear – Articles published prior to 2000 – Non-English language articles – Studies discussing both self-harm and suicide or conflating the two – Studies discussing opinions, definitions or knowledge about self-harm only – Studies exploring a discursive aspect of attitudes towards self-harm, e.g. attributional theories or models, or the factors influencing attitudes – Studies comparing attitudes towards self-harm, whilst not reporting attitudes to self-harm per se – e.g. comparing attitudes towards one form of self-harm with another or comparing one group of professionals with another – Studies examining non-professional perceptions and attitudes: students, members of the public, people who self-harm or family members

**Figure 1.**
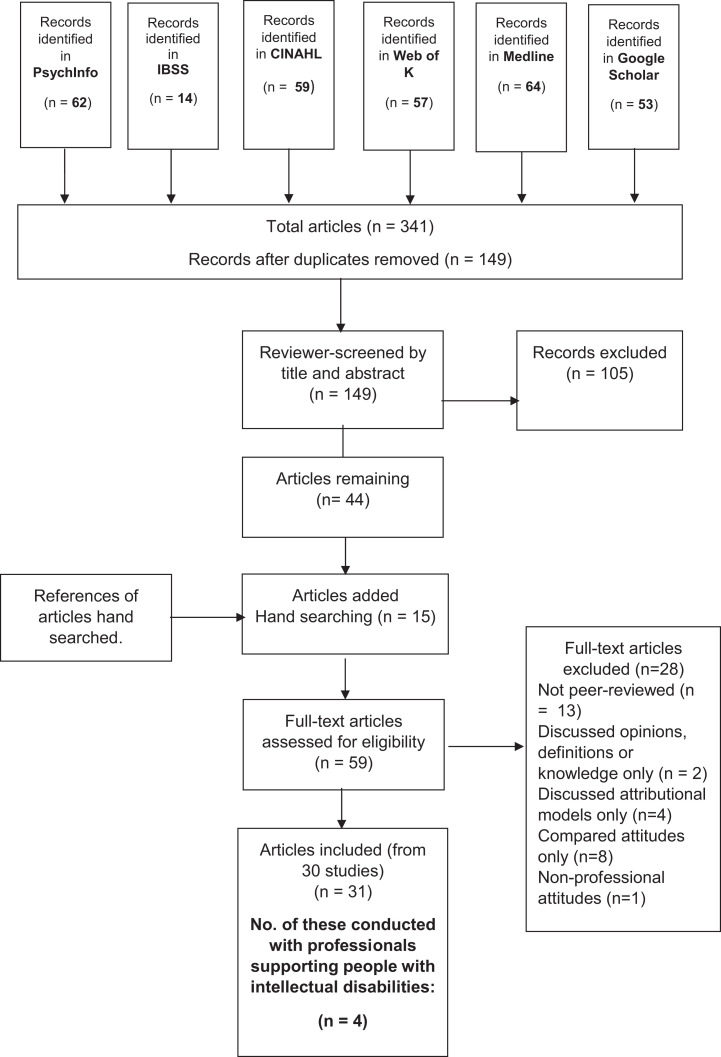
PRISMA flow chart of study identification.

The database search produced 341 articles. The articles were screened for duplications and 192 articles removed. The 149 remaining articles were screened against the inclusion and exclusion criteria resulting in the exclusion of 105 articles. Forty-four articles remained; references of these were searched by hand, producing an additional 15 relevant articles. The full text articles for the resultant 59 articles were assessed for eligibility against the inclusion and exclusion criteria. Thirty-one articles from 30 studies were included at the final stage; 4 of these related specifically to people with intellectual disabilities and are the focus of this article. This process is illustrated in Figure 1.

### Analysis

Study data was extracted from the full text articles, capturing author, year, location of study, aims, methods and analysis, role and number of participants (or number of studies) and key findings. The studies with professionals in services supporting people with intellectual disabilities were analysed as a discreet group. The findings relating to attitudes towards self-harm were extracted from each article, and these findings repeatedly read, so that themes were identified iteratively (Robertson et al., 2019). This informed the identification of five themes: knowledge of staff members; training and education; characteristics of professionals (which were explored as relating to their attitudes); attitudes; and recommendations.

The results are presented utilising narrative synthesis. The studies were divided into Group A: professionals supporting people with intellectual disabilities who self-harm and Group B: professionals supporting people who self-harm in settings not specific to people with intellectual disabilities. This article gives particular focus to the findings from the four papers examining attitudes (including attributions) of professionals supporting people with intellectual disabilities (Group A). The findings from Group B are summarised briefly and contrasted with the findings from Group A.

## Results

Four articles were found that researched professionals’ attitudes and attributions towards people with intellectual disabilities who self-harm (Dick et al., 2011; Fish, 2000; James and Warner, 2005; Snow et al., 2007). All four articles were UK-based research with both qualified and unqualified care staff. The studies used a variety of methodologies: two studies utilised Q-methodology (Dick et al., 2011; James and Warner, 2005); Fish (2000) conducted in-depth interviews and Snow et al. (2007) conducted a questionnaire study. They were from a range of settings: Dick et al. (2011) conducted their research within community services; Fish’s (2000) research was in a forensic intellectual disability service; James and Warner’s (2005) research was based in a medium secure unit; and Snow et al. (2007) study was conducted in in-patient services for people with intellectual disabilities.

The four papers situate themselves variably in terms of the conceptualisations of ‘self-harm’, the terms they use and the theoretical framework in which they are positioned. James and Warner (2005) introduce their paper with a discussion about definitions of self-harm in relation to those with intellectual disabilities. They argue that ‘because “intent” is often difficult to establish’ for people with intellectual disabilities ‘the focus is shifted from a concern with function or motivation to the act itself’ (p. 121). They contend that this potentially explains why self-harm amongst those with intellectual disabilities is often considered ‘challenging behaviour’, which ‘reinforce[s] the notion that people with learning disabilities act without reason’ (p. 121). Their adoption of the term ‘self-harm’ is part of an explicit commitment to the notion that ‘different understandings will make sense for different people at different times and in different situations’ (p. 122): i.e. they resist the separation of self-harm for those with and without intellectual disabilities. Similarly, Dick et al. (2011) begin their article with an assertion that people with intellectual disabilities ‘may self-harm for the same reasons’ (p. 233) as people without intellectual disabilities, situating their research amongst literature arguing ‘for a broader understanding of self-harm than that offered by behavioural and biological theories’ (p. 234). Fish (2000) situates her study in reference to research with people with and without intellectual disabilities, not discussing the difference in conceptualisations, and using the terms ‘self-harm’ and ‘self-injury’ interchangeably. Snow et al. (2007) use the term ‘self-injurious behaviour’, incorporating this within the wider concept of ‘challenging behaviour’. They make no reference to self-harm in the general population and situate themselves within the behavioural literature. All four papers discussed staff’s understandings of self-harm, their attributed reasons for self-harm and, whether implicitly or explicitly, their attitudes towards it.

Twenty-six studies (27 articles) were conducted in settings which supported people predominantly without intellectual disabilities and formed Group B; they included studies with professionals working in education services (4 articles), general healthcare services (13 articles), mental health services (5 articles) and prison services (5 articles). There was a wide variety of methods, with 15 questionnaire studies, using a mix of quantitative and qualitative analysis, 5 qualitative studies and 6 literature reviews or systematic reviews.

### Thematic analysis of Group A papers

Three major themes around attitudes and attributions were identified in the four studies conducted with professionals working with people with intellectual disabilities. Professionals understood self-harm as:

1. having an individual and complex meaning relating to:
  - distress connected to previous experiences,
  - distress connected with the here and now;
2. a way of communicating something;
3. a product of the person’s internal state.

Three of the four studies (Dick et al., 2011; Fish, 2000; James and Warner, 2005) found that staff understood self-harm as ‘*having an individual and complex meaning that is likely to be associated with distress*’ (Dick et al., 2011: 236). The studies reported that staff thought self-harm was commonly a way to cope with internal difficulties, such as powerlessness and abuse or the struggle to process difficult experiences. It was felt to operate like a ‘*safety valve through which they may release tensions and emotions*’ (James and Warner, 2005: 124). Similarly, Fish (2000) reported that staff understood the reasons for self-harm ‘*to be individual coping strategies*’ (Fish, 2000: 205). Dick et al. (2011) found a relatively high level of agreement with the notion that self-harm was individual and was *‘emotionally meaningful*’ (p. 236); they concluded that staff understood the complex and individual meaning for self-harm and that it was a way to *‘cope with and communicate distress’* (p. 236).

All four studies found that staff understood self-harm as a way in which clients coped with the here and now: it was considered a way to manage immediate, external circumstances (Dick et al., 2011; Fish, 2000; James and Warner, 2005; Snow et al., 2007). For instance, Fish (2000) reported that staff felt that the constraints of the environment and the sense of loss of control meant that normal techniques for managing difficult emotion were not available to clients, and self-harm became a way to cope in the moment. James and Warner (2005) concurred, whilst specifying that whilst the staff account understood ‘*self-harm [as] a way of coping with their current situations, it does not consider that the situation* per se *causes the women to self-harm*’ (p. 123). Similarly, Dick et al. (2011) reported that staff viewed self-harm ‘*as an understandable way to communicate distress*’ (p. 237).

Secondly, in all four studies, self-harm was viewed as a way of communicating, as having a perceivable ‘function’, as functional analysis might explicate. Self-harm was acknowledged as ‘*both a response to and an attempt to influence the behaviour of others*’ (Dick et al., 2011: 238). James and Warner (2005) similarly found that self-harm was understood as adaptive and meaningful. Fish (2000) reported that staff felt that self-harm gave the client something of which to be in control, or a way to express their loss of control: it was a way to cope or a way to rebel against feeling controlled or lacking control. Some staff interpreted this as manipulative behaviour. Similarly, staff in Snow et al. (2007) study reported that self-harm was directed to affect a particular outcome, although attributing self-harm to needing or wanting attention was also associated with higher levels of emotional exhaustion in the staff.

Both Fish (2000) and Dick et al. (2011) discussed as a critical theme the complexity of the relationships between staff and clients; it was within the context of these relationships that self-harm was used to communicate. For three of the four studies, self-harm was interpreted as ‘*meaningful within the context of relational interactions*’ (Dick et al., 2011: 238). This also included the idea that someone might self-harm to copy other people. This was interpreted as *‘an attempt to identify with others and thus build relationships’* (Dick et al., 2011: 238).

Thirdly, self-harm was also associated with the person’s internal state, as articulated by a participating staff member in Fish’s (2000) study: ‘*that’s part of her, part of her personality*’ (p. 201). Snow et al. (2007) found that self-harm was largely attributed to internal factors that originate with the person that are beyond their control (i.e. their personality or their intellectual disability). Dick et al. (2011) found that some staff felt that self-harm was specific to having intellectual disabilities: ‘*to some extent this viewpoint suggests that self-harm is lacking intent or meaning when carried out by people with learning disabilities*^3^’ (p. 237). However, there were some contradictions and complexities surrounding this idea, with staff also reporting that they felt that self-harm could be a way to communicate abuse or distress. In this sense, Dick et al. (2011) found *‘a split between viewing self-harm as a meaningless response to biological factors and acknowledging that self-harm can be an attempt to communicate distress associated with experiences of abuse and being dissatisfied with life circumstances’* (p. 238). James and Warner’s (2005) study was the only one to rebuff this explanation outright, reporting that staff rejected *‘the notion that self-harm is a learned behaviour, either from childhood or through institutionalization’* (p. 124).

All the studies found a mixture of contradictory attributions and attitudes. There were some contradictions and complexities within staff’s attitudes and attributions, with self-harm understood both as adaptive and meaningful – a way to manage distress connected with previous and current experiences, or a way to communicate something – but also at times intrinsically part of the person (Dick et al., 2011; James and Warner, 2005).

### Summary of thematic analysis of Group B papers

Three primary themes were found in the group B papers amongst professionals supporting people in settings not specific to people with learning disabilities. Firstly, self-harm was understood as emotionally meaningful: a way to regulate emotion (Berger et al., 2014; Crawford et al., 2003) or an expression of distress (Conlon and O’Tuathail, 2012; Freidman et al., 2006; Koning et al., 2018; Pannell et al., 2003); the studies typically linked this attribution as relating to empathetic and compassionate attitudes. Secondly, by staff who felt ill-equipped, frustrated or helpless, self-harm was more likely to be described as attention-seeking and manipulative (Bhola and Ravishankar, 2017; Dickinson and Hurley, 2012; Hodgson, 2016; Marzano et al., 2015; Sandy and Shaw, 2012; Saunders et al., 2012; Short et al., 2009). Thirdly, staff ascribed self-harm to a person’s mental health issues (Bhola and Ravishankar, 2017; Heath et al., 2006; Marzano et al., 2015; Pannell et al., 2003). These themes are expounded in contrast to the themes found in the four studies examining the attitudes of professionals working with people with intellectual disabilities.

The summary of themes from Group A and B can be viewed in Table 3.

**Table 3.** Summary table of themes.

Themes from Group A	Themes from Group B
1. Self-harm had an individual and complex meaning relating to:	1. Self-harm was emotionally meaningful – a way to regulate distress, to cope or escape from difficult feelings.
a) distress connected to previous experiences;	
b) distress connected with the here and now.	
2. Self-harm was a way of communicating.	2. Self-harm was a way to get attention or manipulate others.
3. Self-harm was a product of the person’s internal state.	3. Self-harm was a product of someone’s mental illness.

### Contrasting the findings

A key theme of the four papers in Group A was that self-harm was individual and complex, and a way to cope with distress. This distress was perceived as connected with previous experiences – e.g. powerlessness, abuse or the struggle to process difficult experiences – as well as difficult experiences connected with current circumstances.

Most staff in the Group B studies also reported that self-harm was emotionally meaningful: ‘[it’s] a release when they can’t deal with emotional turmoil’ (Berger et al., 2014: 205). This was described variably as a form of emotional regulation (Berger et al., 2014; Crawford et al., 2003), an expression of distress (Conlon and O’Tuathail, 2012; Freidman et al., 2006) or an attempt to cope (Koning et al., 2018; Pannell et al., 2003). This aligned with the notion of self-harm being perceived as individual and complex and connected to difficult emotions connected with previous and current experiences, as found in Group A papers (Dick et al., 2011; James and Warner, 2005). In short, the conceptualisation of self-harm as an expression of distress, whether about previous or current experiences, was found amongst professionals supporting people with and without intellectual disabilities. This view aligns itself with the psychosocial perspective of self-harm: that it is a response to emotional distress.

The second theme identified was that self-harm was reported as a form of communication – as having a function. This was broadly found in both groups of studies and most professionals appeared to have some understanding that self-harm was sometimes an attempt to gain something needed or change something about a person’s situation. However, for professionals working with people without intellectual disabilities, this was viewed much more negatively.

In Group B studies staff attributing self-harm to a person trying to communicate was often reported as an unhelpful or unconstructive attribution; this is because staff normally expressed this from a place of frustration or anger, labelling communicative self-harm ‘to get attention’ or ‘manipulative’ (e.g. Bhola and Ravishankar, 2017; Dickinson and Hurley, 2012; Marzano et al., 2015; Sandy and Shaw, 2012). Sometimes, frustration about this sort of self-harm led to staff making judgements about whether self-harm was ‘genuine’ or ‘not-genuine’ (Conlon and O’Tuathail, 2012; Hodgson, 2016; Saunders et al., 2012). These judgements were often made according to whether a person was perceived as being ‘in control’ of the behaviour (and by implication using it for gain) or ‘not in control’ (could not help it and was therefore more worthy of sympathy and support) (Short et al., 2009). For staff in Group B studies, self-harm functioning as a way to communicate or achieve something relationally was often unacceptable.

However, this function was generally considered an understandable reason to self-harm in the four papers in the Group A studies: professionals understood that a person might self-harm to gain something in the moment – attention, escape, a change in sensory input or something physical (Fish, 2000; Snow et al., 2007). Communicative self-harm was understood as meaningful within the context of relationships (Dick et al., 2011; Fish, 2000), and professionals were largely working from an assumption that relationships were significant to their work and a critical element that interacted with service user’s self-harm. This apparent ease in considering the importance of relationships allowed for a reframing of the attributions that were viewed negatively in Group B studies. For instance, James and Warner (2005) identified the theme ‘coping with the here and now’, within which was situated that some staff felt self-harm was an attempt to influence ward staff (p. 124); this would more typically have been themed under ‘attention-seeking’ or ‘manipulation’ by the Group B studies, whereas James and Warner (2005) concluded instead that it suggests ‘the focus, therefore, is on external relationships’ (p. 124). Dick et al. (2011) similarly reframed the factors which might suggest self-harm was manipulative, naming it ‘self-harm within the context of relationships’ (p. 238).

The third theme identified in the papers about self-harm in people with intellectual disabilities was that self-harm occurred because or in relation to the presence of intellectual disabilities (Dick et al., 2011; Snow et al., 2007). Dick et al. (2011) asked participants to rate the statement: ‘people with learning disabilities self-harm because of their learning disabilities’ (p. 242); to which there was low agreement. Similarly, James and Warner (2005) asked participants to rate the statement: ‘women with learning disabilities who self-harm are displaying stereotyped behaviour’ (p. 127). These are not statements found in the Group B studies, as they are both based on theories advocated in the intellectual disabilities literature. However, there was an equivalent attitude: if a person self-harms because they have intellectual disabilities, then a person without intellectual disabilities might be similarly framed as self-harming because they have a mental health illness. These statements effectively place the locus of the self-harm inside the person (Huband and Tantam, 2000): something in the person makes them self-harm – it’s how they are or who they are. This broader notion – that self-harm was a product of a person’s biology – was found in some of the Group B studies (Marzano et al., 2015; Pannell et al., 2003), though less frequently. Interestingly, for the prison studies, this attribution was also linked to ‘genuineness’ – as it was perceived as non-manipulative and evidence that the person really needed some help, rather than using self-harm for their own gain (Short et al., 2009).

Studies in Groups A and B found that professionals perceived self-harm as having a meaning that was individual to each person and often a means for an individual to manage distress connected with difficult previous or current experiences. Self-harm was also seen as a way to communicate something by professionals in all settings. However, this was couched within the context of relationships amongst professionals supporting people with intellectual disabilities in the Group A studies, in contrast to the negative reporting of communicative self-harm in the Group B studies. Professionals in Group A were more likely than professionals in Group B to attribute self-harm to a person’s biology. Although there was an equivalent attribution amongst the Group B studies, in which self-harm was attributed to a person’s mental health difficulties, the tendency to attribute self-harm in this way was more common in the Group A studies. These comparative findings will be discussed in relation to the wider literature.

## Discussion

Examining staff attitudes towards self-harm is a reasonably well-established field of inquiry, largely prompted by the continual concerns raised about professional attitudes towards self-harm in self-report studies asking people about their experiences. These concerns are reflected amongst those with intellectual disabilities (Duperouzel and Fish, 2010; Griffiths et al., 2013) and without intellectual disabilities (Long, 2018; Owens et al., 2020; Wadman et al., 2018). However, only four studies examining the attitudes of professionals supporting people with intellectual disabilities who self-harm could be found that met the requirements of this review. This is very disappointing in comparison to the 26 studies found examining the same concerns amongst professionals in mainstream settings.

The findings will be discussed in relation to theory and practice, considering the impact of behavioural theories, biological theories and psychosocial theories in turn.

The professionals in Group A studies were more likely to situate self-harm in the immediate circumstances, considering self-harm as a response to a situational or relational factor. This reflects the emphasis on behavioural theory for people with intellectual disabilities who self-harm: i.e. a person self-harms to gain something in the moment – attention, escape, a change in sensory input or something physical (MacLean et al., 2020; Tate and Baroff, 1966). Applied behavioural analysis – the basis of most behavioural interventions – has typically focused on proximal, environmental factors that contribute to self-harm, namely, ‘an examination of what happens before, during and after’ (British Institute of Learning Disabilities (BILD), 2016: 2); this posits that self-harm is ‘positively or negatively reinforced by sensory, tangible or social stimuli’ (Oliver and Richards, 2015: 1045). This is akin to the view that self-harm is responsive to the context of situations and relationships. Professionals within the intellectual disabilities settings appeared to be more comfortable with this notion. The focus on the behavioural aspects of self-harm amongst people with intellectual disabilities, has possibly facilitated professionals to consider the significance of their immediate attitudinal and relational response to a person’s self-harm. Consequently, the studies in Group A had an underlying assumption that they were in a relational setting and doing relational work. This pointed to a different culture and training which accepted that their professional role both involved and required investment in relationships.

A biological model of self-harm has been a secondary feature of theory and practice for people with intellectual disabilities who self-harm. This effectively situates a possible source of the self-harm in the locus of the person – specifically, an aspect of their intellectual disabilities (Bradley et al., 2018; Oliver and Richards, 2015). This attitude towards self-harm – that it is a product of something inside the person – was found in professionals in Group A studies. However, it was also only asked about explicitly by Group A studies. James and Warner (2005) and Dick et al.’s (2011) studies both included statements rooted in biological theory in their Q-methodological studies. Both these studies consciously responded to the biological and behavioural dominant theories found in the sector supporting people with intellectual disabilities. This biological model was also found in Group B studies, though with less frequency, with some professionals attributing self-harm to a person’s mental health difficulties.

There is sometimes concern raised that if professionals situate the locus of self-harm within the person, as a part of their identity, it will lead to a sense of care futility or apathy (Huband and Tantum, 2000); this is because if self-harm is perceived as an intrinsic part of the person, then it potentially negates possible efforts to help them reduce it. This did not appear to be the case amongst the professionals in the Group A studies, who, whilst having some occasions of antipathy and judgement towards self-harm similarly to the Group B studies, also appeared to demonstrate a significant level of thoughtfulness and compassion, which was possibly a reflection of the different capacity for relational work. The fact that biological reasons for self-harm were seemingly never given exclusively may also have mitigated the corresponding potential for feeling hopeless about potential change.

Attitudes reflective of the behavioural and biological models did not dominate the Group A papers. Dick et al. (2011) and James and Warner’s (2005) studies both found that ‘staff beliefs are generally broadening beyond the previously dominant biological and behavioural models’ (Dick et al., 2011: 238). Similarly, Fish (2000) concludes that ‘it is clear that staff in this service perceive the reasons for self-harm to be individual coping strategies, or due to environmental constraints’ (p. 205). The professionals in the Group A studies tended to incorporate the psychosocial model – that self-harm is a response to emotional distress and a struggle to process previous and current difficult experiences – with the biobehavioural model in their responses. There was an understanding that a person with intellectual disabilities might self-harm in response to proximal factors such as behavioural phenotypes and motivation-related interactions (Minshawi et al., 2015; Oliver and Richards, 2015) alongside distal factors such as difficult past or present experiences (Dick et al., 2011; Heslop and Macauley, 2009; Nock, 2009). James and Warner (2005) concluded that the multiple issues perceived to be connected with self-harm ‘calls into question the appropriateness of widely accepted definitions of self-harm in respect to learning disability that emphasize physical damage above intent or function’ (p. 125).

## Conclusion

Self-harm, whether presented by someone with or without an intellectual disability, is concerning. It is an immediate sign that the individual is distressed and needs support; it is also a known risk marker for further mental health problems (Borschmann and Kinner, 2019) and it may reduce the likelihood of friendships and, where relevant, successful placements (Chezan et al., 2017; Minshawi et al., 2015). With the increase in levels of self-harm in the UK (McManus et al., 2019) and the ongoing concerns about the experiences of people with intellectual disabilities who self-harm (Heslop and Lovell, 2013; Richards and Symons, 2018), together with the rising international concern of the potential marginalisation of people with intellectual disabilities in terms of COVID-19 (Clegg, 2020), it has never been more apposite that the response and approach to self-harm is as good as it can be.

The small number of studies in Group A in comparison to Group B, indicates the paucity of research examining attitudes of staff supporting people with intellectual disabilities who self-harm; it is partly impacted by a tendency in the literature to discuss self-harm in amongst wider concerns around challenging behaviour (Chezan et al., 2017; van den Bogaard et al., 2019); indeed, several studies were excluded on these grounds (Bailey et al., 2006; Male, 2003; Wilderjans et al., 2014). This has been highlighted as a disadvantage when wanting to consider people with intellectual disabilities who self-harm without conflating it with other concerns (Chezan et al., 2017; Minshawi et al., 2015). However, even accounting for the studies excluded on these grounds, there was simply less research available that met the criteria for Group A, highlighting the need for more research in this area.

The differences found between attitudes of professionals supporting people with and without intellectual disabilities who self-harm related to current theory, with professionals supporting people with intellectual disabilities more likely to attribute self-harm in line with the dominant biobehavioural model. However, a relational emphasis was also found in the Group A studies, which reframed some of the more ‘manipulative’ attributions of self-harm within the context of the relationships between staff and service users. This was something that the professionals in Group B studies struggled with, instead expressing frustration about ‘manipulative’ aspects of self-harm. Further research should explore these differences between the sectors, building on the comparative aspect of this review. It may be that the relational emphasis found amongst professionals supporting people with intellectual disabilities, and which aligns with NICE Guidance on Self-Harm (2013) more comfortably, might lend itself to multi-disciplinary training and practice-sharing.

There was evidence that professionals supporting people with intellectual disabilities expressed attitudes and attributions towards self-harm that were reflective of both the biobehavioural theory, which has typically dominated practice with people with intellectual disabilities, and psychosocial theories, which has dominated practice with people without intellectual disabilities who self-harm. Research and practice with people with intellectual disabilities who self-harm should continue to explore the incorporation and application of both sets of theory and practice, as both have utility.

## Notes

1. Maintenance is the term used in the literature to mean the on-going use of self-harm (Oliver and Richards, 2015).

2. This coincides with the introduction of NICE Guidance on Self-Harm (2013).

3. This study uses the term ‘intellectual disabilities’ throughout, except when direct quotations use alternate terms.
